# Establishment of Chimerism and Organ Transplant Tolerance in Laboratory Animals: Safety and Efficacy of Adaptation to Humans

**DOI:** 10.3389/fimmu.2022.805177

**Published:** 2022-02-10

**Authors:** Robert Lowsky, Samuel Strober

**Affiliations:** ^1^ Division of Blood and Marrow Transplantation and Cancer Cellular Therapy, Stanford University School of Medicine, Stanford, CA, United States; ^2^ Division of Immunology and Rheumatology, Stanford University School of Medicine, Stanford, CA, United States

**Keywords:** chimerism, transplant tolerance, immune suppression, animal models, human trials

## Abstract

The definition of immune tolerance to allogeneic tissue and organ transplants in laboratory animals and humans continues to be the acceptance of the donor graft, rejection of third-party grafts, and specific unresponsiveness of recipient immune cells to the donor alloantigens in the absence of immunosuppressive treatments. Actively acquired tolerance was achieved in mice more than 60 years ago by the establishment of mixed chimerism in neonatal mice. Once established, mixed chimerism was self-perpetuating and allowed for acceptance of tissue transplants in adults. Successful establishment of tolerance in humans has now been reported in several clinical trials based on the development of chimerism after combined transplantation of hematopoietic cells and an organ from the same donor. This review examines the mechanisms of organ graft acceptance after establishment of mixed chimerism (allo-tolerance) or complete chimerism (self-tolerance), and compares the development of graft versus host disease (GVHD) and graft versus tumor (GVT) activity in complete and mixed chimerism. GVHD, GVT activity, and complete chimerism are also discussed in the context of bone marrow transplantation to treat hematologic malignancies. The roles of transient versus persistent mixed chimerism in the induction and maintenance of tolerance and organ graft acceptance in animal models and clinical studies are compared. Key differences in the stability of mixed chimeras and tolerance induction in MHC matched and mismatched rodents, large laboratory animals, and humans are examined to provide insights into the safety and efficacy of translation of results of animal models to clinical trials.

## Introduction

Currently, about 500,000 patients with end stage renal disease are undergoing dialysis in the US of which over 100,000 are on a wait list for a deceased or living donor transplants (1). In addition, there are patients who have yet to start dialysis and require a kidney transplant for end stage renal disease. The treatment of choice for these patients is kidney transplantation, since transplantation allows the best chance for a return to a near normal life style, and an improved life expectancy (2–4). Recipients of living and deceased donor kidneys require strict and lifelong adherence to combinations of immune suppression (IS) medications to prevent immune mediated rejection of the transplanted kidney. Most commonly, recipients require a two or three-drug IS regimen consisting of a purine metabolism inhibitor, a calcineurin inhibitor, and steroids (5, 6).

However, even with the use of these potent drug combinations there is continued and progressive loss of the kidney graft due to immune mediated graft rejection: median graft survival of living donor HLA mismatched kidneys is ~15 years, and recipients of deceased donor kidney transplants have graft survival averaging 8-12 years (7–9).

Long-term patient survival after graft loss is poor. In addition to graft rejection, the IS drug regimens themselves cause significant medical comorbidities that include chronic allograft vasculopathy, new onset diabetes after transplant (NODAT), infections, cancer, heart disease, hypertension, renal dysfunction, and osteoporosis and osteopenia (10–13). It has been shown that the number of IS medications per day and the occurrence of IS-related adverse effects have tremendous impact on a patient’s quality of life and adherence to treatment (10, 11). Moreover, dosage adjustments to reduce the IS medications have been associated with acute rejection (10, 11). Patient noncompliance with IS drug regimens is the third leading cause of graft loss in renal transplantation, after chronic allograft nephropathy and death with a functioning graft (14). Both chronic allograft nephropathy and death with a functioning graft are related, directly and indirectly, to chronic IS drug regimens. Thus, the main limitations to successful and safe organ transplants are immune mediated graft rejection, and the medical comorbidities induced by the combinations of IS medications that help deter or delay but not prevent rejection. Strategies that result in IS drug minimization and/or complete withdrawal while maintaining normal graft function would represent a significant health benefit to patients undergoing transplantation. The unmet medical need is to eliminate the lifelong requirement of IS drug combinations with their attendant side effects, and to prevent immune mediated rejection of donor organ transplants. From pioneering studies of more than 70 years ago, it is long known that the establishment of hematopoietic cell chimerism in organ transplant recipients would lead to normal graft function without IS medications and without evidence of histologic rejection. This review will concentrate on preclinical models of chimerism-based transplantation tolerance, and highlight the bench-to-bedside adaptation of the models by combining living donor kidney and hematopoietic cell transplantation to purposefully withdraw IS medications from patients while maintaining normal graft function.

### Definitions of Mixed Chimerism, Complete Chimerism, and Immune Tolerance to Organ Transplants

Seminal observations of the survival of skin grafts in adult dizygotic cattle that shared a placenta *in utero* with the skin graft donor, and in adult mice that had been given injections of genetically disparate bone marrow cells from the skin graft donor strain during the fetal/neonatal period led to the concept of “tolerance” as opposed to the expected “rejection” of the grafts (15–17). The specificity of the tolerant state was demonstrated by the ability of the recipients to reject third party but not donor skin grafts (16, 17).

Tolerance was linked to “genetical chimerism” in these skin graft recipients, and the term was used to mean that two or more cell lineages with different genotypes (allogeneic) were present in the recipients (15–17). Subsequent studies of erythrocyte chimeric cattle twins showed that skin grafts exchanged between them had prolonged survival, but were eventually rejected (18). In this model system, immune rejection was attenuated, and not completely prevented. Thus, the skin graft recipients did not meet the criteria of tolerance that includes lack of evidence of rejection of the donor graft.

At present the term “mixed chimerism” is used to identify recipients with a mixture of two or more hematopoietic and immune cell lineages in the blood forming and lymphoid tissues (19–21). Numerous preclinical studies have shown that the recipient and donor immune cells of mixed chimeras show a mutual state of immune tolerance (specific unresponsiveness) to donor and recipient alloantigens, but not to third party alloantigens (18–21). The term “mixed chimerism” is currently used to distinguish human or non-human recipients from those with “complete chimerism”.

Studies with complete chimerism in humans involve recipients who were treated with blood and marrow transplants as curative therapy for a hematologic malignancy. In these patients the donor hematopoietic and immune cells completely replaced the recipient hematopoietic and immune cells (22–25). The development of complete chimerism is desirable to prevent tumor relapse because complete chimerism associated with beneficial alloreactive graft-versus-host reactions necessary for immune mediated eradication of residual host-derived cancer cells. However, complete chimeras have a higher risk of developing harmful graft versus host disease (GVHD) as compared to mixed chimeras (25, 26). The risk of GVHD in human complete chimeras has been mitigated even in HLA mismatched transplant patient pairs by the development of several strategies including the administration of posttransplant cyclophosphamide (PTCy) (27, 28).

Reports of cancer patients with complete chimerism after bone marrow transplantation and who thereafter developed end stage renal disease (ESRD) accepted a kidney transplant from their bone marrow donor, and without the need for immunosuppressive (IS) drugs (29, 30). The acceptance of the organs in the latter recipients did not reflect a proof of concept of “immune tolerance”, rather these cases represent acceptance of “self” histocompatibility antigens shared between the cells of the donor organ and the cells of the donor bone marrow, since there were only chimeric donor immune cells and no residual detectable recipient immune cells in the blood or marrow (29, 30). Complete chimeras can develop donor immune cells that are specifically unresponsive to recipient alloantigens (31, 32). This represents graft versus host (GvH) acceptance. The use of pretransplant host conditioning using total lymphoid irradiation (TLI) combined with anti-thymocyte globulin (ATG) or the administration of cyclophosphamide shortly after bone marrow transplantation in laboratory animals and in humans has been shown to establish GvH acceptance that prevents GVHD (27, 32, 33).

## Studies of Tolerance in Laboratory Animals

### Establishment of Mixed Chimerism and Tolerance to Skin and Organ Grafts in Neonatal Rodents: Stability of Chimerism in the Absence of Immunosuppressive Drugs

The seminal observations of the development of stable mixed chimerism in cattle that shared a placenta *in utero*, and the inability of the adult chimeras to rapidly reject skin grafts from each other, but not from third parties, formed the conceptual framework for the linkage of chimerism and tolerance (15–17). The key advance was made after the intentional establishment of chimerism and tolerance by injecting neonatal mice of one strain with bone marrow cells from another (34, 35). As in the case of the cattle, the adult mixed chimeras accepted skin grafts from the marrow donor strain but not from third party strains (34, 35). Follow up studies showed that recipient immune cells obtained from the chimeras were specifically unresponsive to the alloantigens of the donor strain and not to third party strains (35–37). Thus, the recipients met the current definition of tolerance by the acceptance of donor grafts, rejection of third-party grafts, and evidence of specific immune unresponsiveness of recipient immune cells to donor antigens (19–21). This is referred to as “host versus graft (HvG) tolerance” or “organ transplant tolerance”. As in the case of the chimeric cattle, mixed chimerism was self-perpetuating in the mice, and levels of chimerism remained stable during adulthood in absence of IS drugs (38, 39). Occasionally the injected neonates showed a phenomenon called “runting” that upon further investigation was found to be a consequence of GVHD after the development of complete instead of mixed chimerism (16, 40). The development of GVHD is an indication that GvH tolerance failed. Mixed chimeras develop bidirectional HvG and GvH tolerance.

### Establishment of Chimerism and Acceptance of Skin Grafts after Myeloablative Lethal Total Body Irradiation of Adult Rodents

The application of the principles of induction of tolerance after the establishment of chimerism in neonates was initially applied to adult mice conditioned with a lethal dose of total body irradiation (TBI). Irradiated adults from one parental strain were given F1 hybrid MHC matched bone marrow transplants followed by skin grafts from the allogeneic parental strain (34). The use of F1 hybrid marrow avoided the development of GVHD, since the immune cells in the marrow were naturally unresponsive to the tissues of the recipient due to sharing of genetically determined histocompatibility antigens with the recipients. The key observation was that the allogeneic skin grafts were accepted after transplantation of hybrid but not syngeneic marrow (34). Although the studies of irradiated adults shared similar observations of skin graft acceptance with that of studies of graft acceptance in neonates, the radiation chimeras given myeloablative lethal TBI were likely to be complete chimeras rather than mixed chimeras due to depletion of recipient immune and hematopoietic cells, and the complete replacement by donor immune and blood forming cells. Thus, the acceptance of the skin grafts in the adults was not due to residual recipient cells developing immune tolerance to donor alloantigens, but rather due to a failure of the donor hybrid immune cells to reject an organ expressing the alloantigens of the shared parental strain (34).

Subsequent studies of acceptance of skin transplants in complete chimeras given lethal TBI, and bone marrow transplants from fully allogeneic strains (donor parental strain cells instead of F1 hybrid cells) also showed acceptance of allogeneic skin grafts in the chimeras (35). However, a proportion of the recipients developed severe GVHD depending on the level of MHC matching (35). The studies of radiation chimeras and acceptance of skin grafts by complete chimeras were of considerable interest. However, acceptance of organ grafts was based on the failure of the chimeric donor cells to reject donor organ grafts. This failure can be explained by the concepts of the lack of donor immune cell responses to donor self-molecules initially described by Burnet, and not on the concepts of immune tolerance to alloantigens initially described by Medawar and his co-workers [reviewed in (41)]. As pointed out in the latter review, experiments of the Medawar group were interpreted as showing that “fully tolerant recipient mice could be said to show ‘central failure’ of their own response to the tolerated transplantation antigens”. These experiments did not prove that purified chimeric recipient immune cells were specifically unresponsive to donor alloantigens, since the technology to perform such experiments did not exist at that time. Subsequent experiments using adult murine mixed chimeras that were tolerant of organ grafts proved that purified chimeric recipient immune cells were unresponsive to donor alloantigens in association with clonal deletion (see below).

### Establishment of Mixed Chimerism and Immune Tolerance to Skin Grafts After Non-Myeloablative TLI of Adult Mice

The risks of myeloablative radiation and of GVHD after allogeneic bone marrow transplantation were justified for adaptation to humans treated for hematologic malignancies, since these diseases were uniformly lethal (22, 42). These risks were mitigated by the use of fully MHC matched transplant pairs that were studied extensively in dogs prior to the application to humans (43). However, the use of lethal TBI described in the studies of myeloablative radiation mouse chimeras discussed above to achieve acceptance of organ transplants could not be applied to humans undergoing organ transplant surgery due to the inherent dangers of lethal irradiation in the recipients, and the development of GVHD.

An alternative was to condition recipients of combined organ and bone marrow transplants with non-myeloablative radiation regimens that had been proven safe in humans. Accordingly, the laboratory of Strober and his co-workers at Stanford University studied the non-myeloablative radiation regimen of TLI for application to pre- clinical models of bone marrow transplantation with or without organ transplantation (38, 39, 44–52). The TLI radiation regimen was developed by Kaplan, Rosenberg, and their Stanford co-workers for the treatment of patients with early stage Hodgkin’s disease (HD) [reviewed, (53)]. TLI radiation was given as multiple small doses over several weeks targeted to the spleen, lymph nodes above and below the diaphragm, and thymus of HD patients (53). All other non-lymphoid tissue areas including the lungs, central nervous system, intestines etc. were shielded with lead. About 50% of the marrow volume was shielded and prevented the development of severe neutropenia and thrombocytopenia observed with myeloablative TBI. TLI was successful in the induction of durable complete remissions in early stage HD, and long-term studies of safety and efficacy were made on thousands of patients starting in the 1960’s (53).

A TLI regimen was developed for use in mice and rats by manufacture of a lead jig that allowed for the irradiation of the spleen, thymus, and lymph nodes and shielded the lungs, skull, liver, portions of the intestines, and marrow in the limbs (38, 39, 44–46). Unexpectedly, when adult TLI treated recipient mice were given bone marrow transplants from fully MHC mismatched donor mice stable self-perpetuating mixed chimerism was established without evidence of GVHD (38, 39, 44–46). In contrast, mice conditioned with a single dose of myeloablative TBI and given mismatched bone marrow transplants uniformly died of GVHD (38, 39, 47, 48). In view of the success in achieving persistent mixed chimerism without GVHD in adult mice, TLI treated mixed chimeras were given skin grafts from the marrow donor strain and third-party strains (38, 39, 44). Whereas the donor-type skin grafts were accepted for observation periods of up to 6 months, the third-party grafts were rejected within a few weeks. Purified recipient immune cells collected from the mixed chimeras were shown to be specifically unresponsive to donor alloantigens in the mixed leukocyte reaction, and clonally deleted to the alloantigens (49, 54). Thus, the recipient mice fulfilled the criteria of immune tolerance discussed above, using a safe non-myeloablative regimen without GVHD that could be applied to adult humans (32). Subsequent to the studies of transplant tolerance with mixed chimerism in rodents and dogs by the group at Stanford, Sachs and his co-workers at Harvard, developed a regimen to achieve stable mixed chimerism and immune tolerance without GVHD in MHC mismatched adult mice by injecting a combination of recipient and donor bone marrow cells into recipients conditioned with TBI (55, 56). Thus, establishment of mixed chimerism was clearly linked to the induction of tolerance in adults in this model also. The contribution of clonal deletion of donor-reactive recipient T cells to the development of tolerance in these mixed chimeras was demonstrated in subsequent experiments (54).

### Establishment of Mixed Chimerism and Immune Tolerance to Heart Transplants in Adult Rats Using a Completely Posttransplant TLI Conditioning Regimen

**Figure 1:**
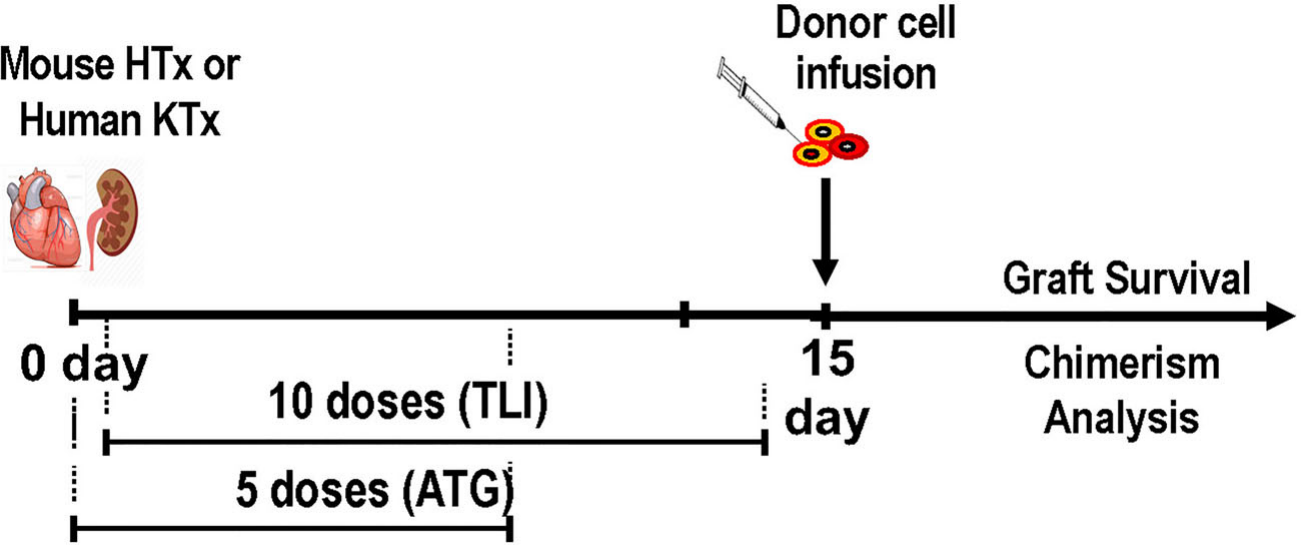
Experimental Scheme for Establishment of Mixed Chimerism and Tolerance in Laboratory Animals and Patients After Organ Transplantation; Donor organ transplantation (heterotopic heart in mice, kidney in humans) is performed on day 0, and the first of 5 daily doses of ATG is given on day 0. On day 1 the first of 10 daily doses of TLI is given. Animals receive 5 doses during the first week and 5 doses during the second week posttransplant. Patients receive 4 doses during the first week and 6 during the second week. Donor hematopoietic cells (cryopreserved and thawed in humans, and fresh in mice) are infused immediately after the last dose of TLI. Serial tests of chimerism and organ graft function are performed thereafter.

The TLI regimen developed in mice was adapted for use in rats given combined MHC mismatched bone marrow given intravenously, and heterotopic heart transplants directly anastomosed to the abdominal aorta and vena cava (44). Lead jigs were manufactured to expose the spleen, thymus and lymph nodes as in the mouse model, and 10 doses of TLI with 5 doses of ATG were used for conditioning (44). Initially the conditioning regimen was administered pretransplant as in the mouse model, and mixed chimerism and tolerance were induced in all recipients (44). However, in order to adapt the conditioning regimen for future use in deceased donor organ transplantation in humans, the timing of the TLI/ATG regimen was changed in subsequent experiments, and administered starting one day after rather than 14 days before the organ transplant (**Figure 1**) (57–60). The change was made because of the uncertainty of the timing of the availability of human deceased donor organ, since the start of a pretransplant conditioning regimen cannot be timed according to the organ availability. The infusion of the donor bone marrow cells was performed immediately after the last dose of TLI administered on day 12 posttransplant (57–60). In humans, this would require cryopreservation of donor cells, and thawing at the completion of TLI.

When the completely posttransplant regimen was used, mixed chimerism was established in all rat recipients, and the heart transplants were not rejected during an observation period of up to 6 months (57–60). In further studies, a course of the calcineurin inhibitor, cyclosporine was administered to the rats given the combined bone marrow and heart transplants after the posttransplant TLI/ATG conditioning regimen was completed in order to determine whether posttransplant immunosuppressive drugs would enhance or interfere with chimerism and the induction of tolerance (61). The results showed that the administration of cyclosporine enhanced the establishment of stable mixed chimerism, and tolerance in this model system (61).

Additional experiments were performed to determine whether an infusion of purified donor peripheral blood mononuclear cells (PBMC) or purified blood monocytes could substitute for the infusion of donor bone marrow cells in recipients given heart transplants and TLI/ATG conditioning (58, 59). Although the survival of heart grafts was markedly prolonged after the PBMC or monocyte infusions as compared to that of controls given no donor cell infusion, biopsies of the surviving heart transplants obtained more than 100 days posttransplant showed evidence of moderate to severe chronic rejection on microscopic analysis (58–60). In contrast, control recipients given infusions of donor bone marrow cells showed no evidence of acute or chronic rejection associated with the development of mixed chimerism (60). The results indicated that the latter chimeric recipients were protected from chronic rejection as compared to the non- chimeric recipients given PBMC or monocytes (60).

### Studies of Regulatory Immune Cells Required for the Establishment of Mixed Chimerism and Tolerance in MHC Mismatched Mice Given Combined Bone Marrow and Heart Transplants Using the TLI/ATG Posttransplant Conditioning Regimen

Our further studies used MHC mismatched C57BL/6 (H-2b) and BALB/c (H-2d) mice as either donors or as recipients conditioned with posttransplant TLI/ATG and given combined bone marrow and heart transplants. Four recipient immune cell types were identified that were required for the induction of chimerism and tolerance (47, 48, 61–63). These included CD8+ tolerogenic dendritic cells (DCs), NKT cells, Tregs, and myeloid derived suppressor cells (MDSCs). Deletion of any one of these recipient cells by genetic engineering (ie., Batf-3-/- to selectively delete CD8+DCs and Jalpha18-/- to selectively delete NKT cells) or by administration of depleting mAbs to delete Treg cells and MDSCs abrogated chimerism and tolerance, and add back of these cells restored chimerism and tolerance (47, 48, 61–63).

**Figure 2:**
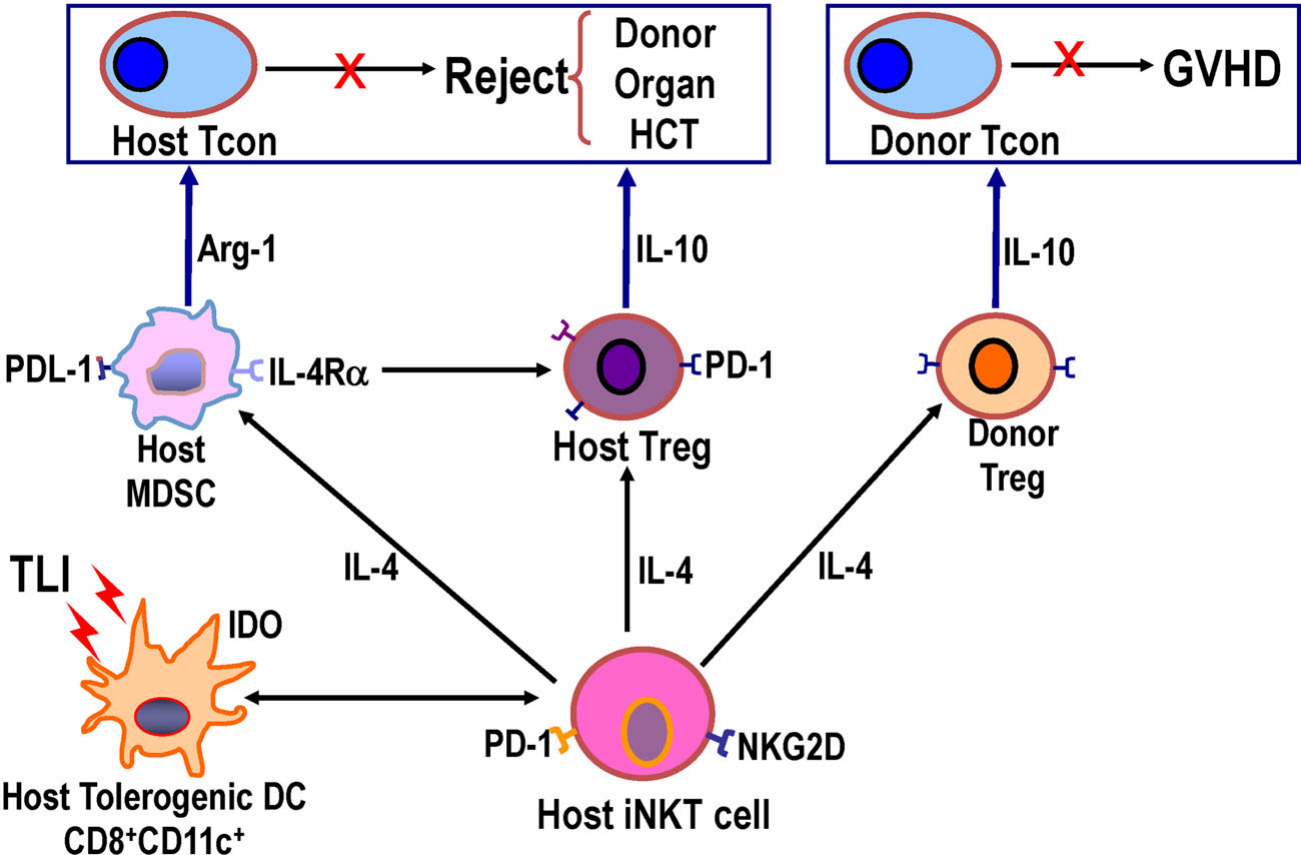
Diagram of network of host and donor regulatory cell interactions after TLI based conditioning regimen and transplantation that are required for establishment of mixed chimerism and tolerance. TLI induced massive apoptosis of lymphocytes, and apoptotic bodies were engulfed by host CD8+DCs that interacted with host NKT cells such that both cell types became immunosuppressive/tolerogenic with NKT cell secretion of IL-4. Host NKT cell interacted with and activated host DCs, host Tregs, host MDSCs, and donor Tregs to upregulate production of immunosuppressive molecules including PDL-1, arginase-1, IDO, IL-10, and PD-1. Knockout or depletion of each of the latter cells or their secreted cytokines abrogated tolerance, and add back of each cell type restored tolerance (62, 63).

The first cells in the chain of interactions in this complex network are Batf-3-/- dependent CD8+DCs that take up apoptotic bodies (efferocytosis) that are produced in great quantities by the TLI procedure (**Figure 2**) (63). The uptake induces changes in the receptors, function, and molecules produced by the CD8+DCs such that they become tolerogenic and express negative signaling surface molecules such as PDL-1 and produce the immunosuppressive cytokines such as IDO (63) The induction of apoptosis is a consequence of the TLI radiation triggering the p53/Bcl2 apoptotic pathway (64, 65). Due to the rapid upregulation of expression of the anti- apoptotic Bcl2 gene in NKT and Treg cells, the balance of these radioresistant T cell subsets is changed to favor these regulatory T cells over radiosensitive naïve Tcon cells (64, 65). The tolerogenic host DCs expressed CD1d and Rae-1 surface receptors that interact with the invariant TCR and NKG2D on host NKT cells such that the latter cells become tolerogenic and secrete abundant IL-4 (63). The NKT cells activate Treg cells in an IL-4 dependent manner as shown in the far left of **Figure 2**, and increase Treg immune suppressive function that is dependent on the secretion of IL-10. Host NKT cells also activated host DCs and MDSCs to become immunosuppressive *via* production of molecules such as IDO, and arginase-1 as reported previously (63, 64). In preclinical models of prevention of GVHD after bone marrow transplantation, Stanford investigators have shown that infusion of donor NKT cells activate donor Treg cells and host Gr-1+ MDSCs, and thereby prevent GVHD (65–67). Analogous changes in the development of immunosuppressive human host MDSCs after TLI based conditioning have been identified (68).

### Tolerance Induction Regimens in Large Laboratory Animals Given Combined Organ and Bone Marrow Transplants

Subsequent to the tolerance studies described above in rodents, several laboratories investigated organ transplantation with bone marrow transplantation using tolerance induction regimens in large animals that were bred and MHC typed in laboratories including dogs, non-human primates, and mini-swine (56, 69–73). Studies in fully MHC matched dogs given non-myeloablative TBI followed by bone marrow transplantation showed that stable mixed chimerism was established for observation periods of at least a few years even after the withdrawal of IS drugs at 6 months posttransplant (69). The mixed chimeras were given donor organ transplants several months after the bone marrow transplants, and almost all recipients accepted the organ without IS drugs without evidence of rejection on biopsies (69). In contrast, third party organ grafts transplanted to the mixed chimeras were rejected rapidly (69). Interestingly, in some experiments the recipient mixed chimeras bearing the donor organ transplants were treated with TBI and recipient leukocyte infusion (RLI) to deplete the donor chimeric cells (73). Despite the loss of chimerism after the RLI, the acceptance of the kidney grafts continued without rejection (73). The results indicated that persistence of mixed chimerism in these fully MHC matched recipients was not required for continued organ graft acceptance (73).

A pretransplant conditioning regimen with non-myeloablative TBI (300 cGy) combined with thymic radiation and ATG allowed for the development of mixed chimerism and acceptance of kidney transplants in MHC mismatched non-human primates given an infusion of donor bone marrow cells at the time of the organ transplant without the need for maintenance IS drugs in the majority of recipients (71, 72). Interestingly the mixed chimerism was transient and lost after a few to several weeks, and the long-term kidney transplants maintained good function without IS drugs despite the loss of chimerism (71, 72). Similar results were achieved in mini-swine given combined MHC mismatched bone marrow and kidney transplants (56). The studies in both the MHC matched dogs and the MHC mismatched non-human primates concluded that transient, but not stable mixed chimerism, was required for organ graft acceptance (70–73). In collaborative studies with investigators at the University of Wisconsin and Stanford University using a TLI/ATG posttransplant conditioning followed by MHC mismatched combined kidney and hematopoietic cell transplantation, long term acceptance of kidney grafts after IS drug withdrawal was also observed after transient mixed chimerism (personal communication, Dixon Kaufmann).

In conclusion, stable mixed chimerism was not required for acceptance of kidney transplants in both dog and non-human primate studies. However, the stability of mixed chimerism in the MHC matched dogs, and the instability in the MHC mismatched non- human primates suggests that there is likely to be a link between stability and MHC matching. This link has not been studied as yet in either of these large animal models, but has been studied in humans (see below).

## Studies of Non-Chimeric Tolerance in HLA Mismatched and Matched Patients

### Feasibility of Non-Chimeric Tolerance Induction in Patients Given HLA Mismatched Kidney Transplants From Deceased Donors Using Pretransplant TLI and Posttransplant ATG Conditioning

Although pretransplant TLI conditioning in fully MHC mismatched mice and rats markedly prolonged skin, heart, and kidney transplant survival, tolerance was not achieved without the addition of a hematopoietic cell infusion and the establishment of mixed chimerism (38, 39, 44, 74–76). In contrast, a study in outbred dogs showed that pretransplant TLI and posttransplant ATG without the infusion of donor cells allowed for acceptance of MHC unmatched heart transplants for more than 1 year in the absence of immunosuppressive drugs (77). The recipients rejected third party transplants and showed specific unresponsiveness to donor alloantigens (77). These recipients showed the feasibility of inducing non-chimeric tolerance to outbred canine kidney transplants using TLI/ATG conditioning.

In view of the results in the canine study, and the safety of the use of TLI to treat patients with Hodgkin’s disease, pretransplant TLI and posttransplant ATG was used to treat a series of patients given HLA unmatched deceased donor kidney transplants to determine the feasibility of maintaining graft function with low dose (0.1- 0.2mg/kg/day) prednisone monotherapy as maintenance therapy (78, 79). TLI was targeted to the lymph nodes, spleen, and thymus with lead shields for all other tissues using multiple doses of 100cGy each given 3 times per week until achieving a total dose of 2,000cGy, and then once per week until the donor organ became available (78, 79). The results showed that the 16 patients who completed the regimen were maintained on prednisone alone at the last observation point with good graft function during a follow up period of up to 25 months. The frequencies of patient survival, graft survival and rejection episodes in experimental patients were similar to that of concomitant standard of care patients maintained on prednisone and cyclosporine, and graft function was improved (78, 79).

In a follow up study, the feasibility of completely withdrawing maintenance prednisone therapy from 3 of the patients given deceased donor kidney transplants and pretransplant TLI was determined (80). The 3 patients showed no evidence of rejection while off IS drugs for 10, 24, and 69 months, and specific unresponsiveness to donor alloantigens was demonstrated in the mixed leukocyte reaction and cell mediated lympholysis assays (80). Thus, the patients met the criteria of actively acquired tolerance. One of these patients was studied again 12 years off IS drugs, and had no evidence of rejection (81). Although, this was the first study to show the feasibility of immune tolerance induction in humans, the frequency of rejection episodes and of graft loss among the experimental patients enrolled in the study who did not develop tolerance was similar to that of concomitant standard of care patients (79). In order to increase the success rate of tolerance induction, and to reduce the frequency of rejection episodes and graft loss, recipients of kidney transplants treated with TLI/ATG conditioning in subsequent tolerance studies were given infusions of hematopoietic progenitor cells from fully or partially HLA matched living donors to establish mixed chimerism (see section 11 below).

### Induction of Non-Chimeric Tolerance in Patients Given HLA Matched Kidney Transplants From Living Donors Using IS Drugs and Infusions of Donor Hematopoietic Cells Without Conditioning

A study of 20 patients who received HLA matched kidney transplants, and multiple injections of donor hematopoietic progenitor cells in order to withdraw maintenance IS drugs was performed by investigators at Northwestern University (82, 83). Recipients were not conditioned with radiation or chemotherapy, and were given 4 infusions of CD34+ selected donor cells along with standard of care IS drugs. The latter included Alemtuzumab induction, short term tacrolimus and MMF that was switched to sirolimus maintenance therapy before complete IS drug withdrawal at 2 years posttransplant on the basis of transplant biopsies at 12,18, and 24 months without evidence of rejection. Peak transient donor chimerism was above 1% (1.7 to 5.2%) in 3 patients, and 5 of 15 patients withdrawn from IS drugs were off drugs without rejection for 60 to 73 months at the last observation point. Seven out of 15 patients withdrawn from IS drugs developed rejection episodes thereafter, and were returned to maintenance therapy (82–84). The patients who maintained good graft function off drugs were considered “operationally “tolerant”, and had increased levels of Treg cells in the blood after transplantation, and a gene signature that was similar to the reported for “operationally” tolerant patients by other investigators (83). Specific unresponsiveness to donor alloantigen as a marker of immune tolerance was not reported.

## Studies of Tolerance in HLA Matched Patients With Mixed Chimerism

### Establishment of Mixed Chimerism and Immune Tolerance in Patients Given HLA Matched Living Donor Kidney and Hematopoietic Cell Transplants

**Figure 3:**
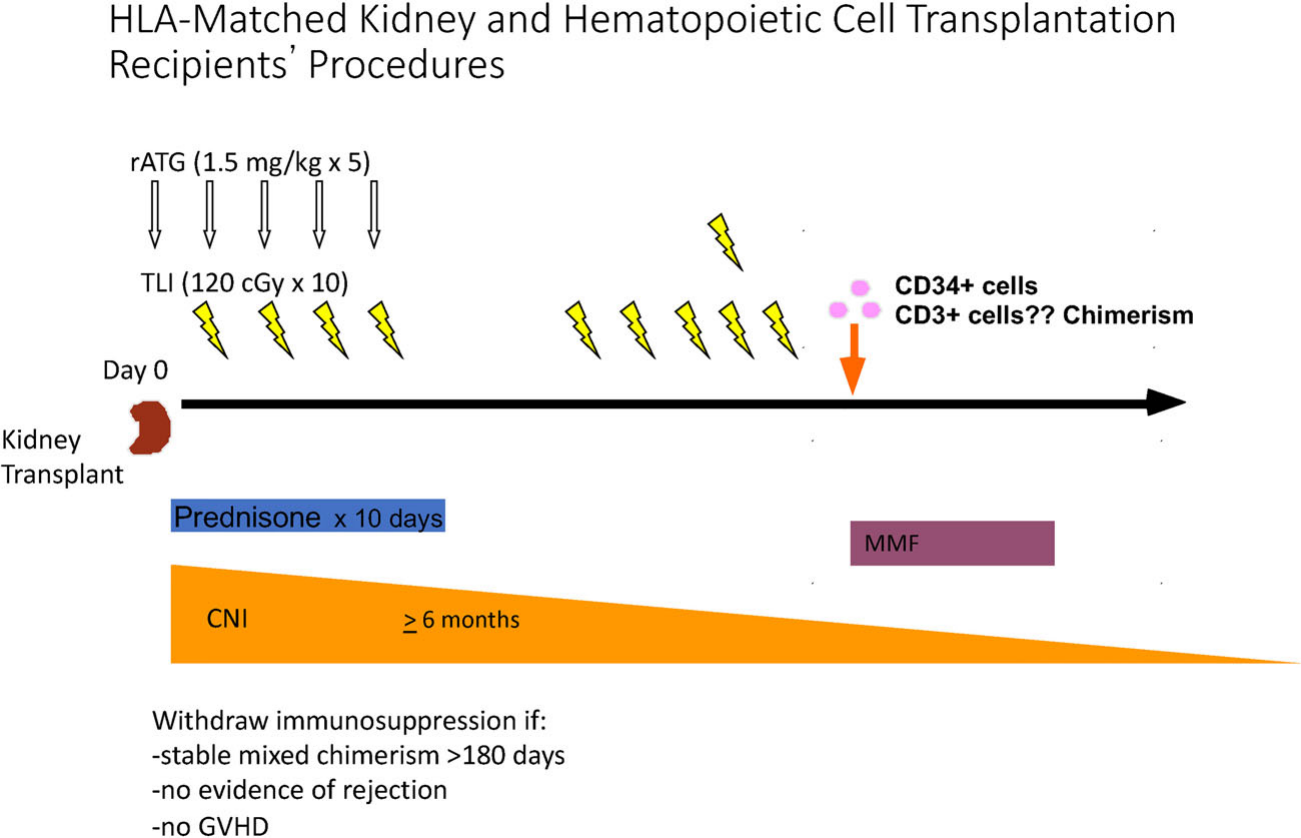
Schema for Establishment of Mixed Chimerism, and Complete Withdrawal of IS Drugs after HLA matched Kidney Transplantation. Kidney transplantation was performed on day 0, and the first of 5 doses of ATG was administered intra-operatively. The first of 10 doses of TLI was administered on day 1, and 3 additional daily doses were administered during the first week. Patients were discharged from the hospital on days 5 or 6 posttransplant, and received and 6 TLI doses in the clinic during the second week posttransplant. Cryopreserved and thawed donor cells were infused immediately after the completion of TLI. Donor cells were collected 2 months before kidney transplantation by apheresis from G-CSF mobilized blood, and CD34 cells were purified. The latter cells and a defined dose of T cells were cryopreserved until infusion after TLI. Prednisone was administered for 10 days, MMF was given for 30 days starting with the day of the donor cell infusion. A calcineurin inhibitor was administered for 6 months posttransplant at standard dosage, and then tapered to discontinuation during the first year posttransplant if chimerism persisted for at least 6 months, there was no evidence of GVHD, and there was no evidence of microscopic rejection on a protocol biopsy performed just before IS discontinuation.

In order to evaluate the ability of TLI/ATG conditioning to promote the establishment of chimerism in humans Stanford investigators first performed a study of allogeneic hematopoietic cell transplants to treat patients with hematologic malignancies (32). In this and in subsequent studies patients received G-CSF mobilized peripheral blood mononuclear cell (PBMC) transplants without CD34+ purification form HLA matched donors who were related or unrelated to the recipients (26, 32). The goal of the latter study was to establish complete chimerism and eradication of tumor cells without GVHD based on preclinical studies that showed that this outcome can be achieved in rodent models using TLI/ATG conditioning (47, 50, 52). Patients enrolled in the study were not considered candidates for myeloablative conditioning due to medical comorbidities and/or advanced age (32). Since persistent chimerism without severe neutropenia or thrombocytopenia was established in almost all the HLA matched recipients with hematologic malignancies (32), a modified protocol using purified CD34+ cells (>4x10^6 cells/kg) and a defined dose of donor T cells (1x10^6 cells/kg) was applied to HLA matched combined kidney and hematopoietic cell transplant patients (**Figure 3**) (85).

HLA matched donor cells were collected, purified, and cryopreserved about 6 weeks before kidney transplantation (85–88). Donor cells were injected intravenously immediately after the completion of 10 doses of TLI. Four doses were given in the first week posttransplant along with 5 doses of ATG. Patients were discharged at day 5 or 6 posttransplant, and the remaining 6 doses of TLI were administered in the out-patient clinics during the second week posttransplant. Twenty-nine patients were enrolled in the study, and 24 who developed mixed chimerism for at least 6 months were completely withdrawn from IS drugs within 1 to 8 months thereafter (85–88). Immune suppression (IS) medications were prednisone for 10 days, MMF for 30 days, and a calcineurin inhibitor (cyclosporine or tacrolimus at standard doses) for six months with gradual tapering to discontinuation thereafter. Withdrawal of IS drugs was dependent of establishment of chimerism for at least 6 months, no evidence of GVHD, and no evidence of microscopic rejection on protocol biopsies just before complete IS drug withdrawal (85–88).

Of the 24 patients withdrawn from IS drugs, 22 had no evidence of rejection with up to 15 years follow up (88). Two patients had rejection episodes at about 4 years after IS drug withdrawal, the episodes were reversed with standard of care treatment, and patients were returned standard maintenance therapy with normal graft function thereafter (88).

Ten patients had mixed chimerism that was persistent until the last chimerism test, and 14 patients lost chimerism sometime after the first year posttransplant (88). Despite the loss of chimerism in 14 of the HLA matched recipients withdrawn from IS drugs, the 12 maintained good graft function without evidence of rejection thereafter, and 2 developed rejection episodes as described above. Patients with mixed chimerism showed specific unresponsiveness of PBMC T cells to donor cells in the MLR, and made normal T cell responses to recall antigens *in vitro*. In summary, the 22 of 29 patients developed mixed chimerism and tolerance to their kidney transplants after combined organ and hematopoietic cell transplantation using TLI/ATG conditioning.

There were no graft loses due to rejection, and no GVHD, severe neutropenia or thrombocytopenia in the 29 patients enrolled in the protocol (85–88).

Medeor Therapeutics performed a multi-center randomized trial of tolerance induction in 20 experimental HLA matched kidney transplant patients and 10 standard of care patients based on the results of the clinical study report by investigators at Stanford University (89). Experimental patients were conditioned with posttransplant TLI/ATG, and given an infusion of donor CD34+ enriched cells and a defined dose of T cells to establish persistent mixed chimerism (89). The interim analysis showed the ability to achieve persistent chimerism in all enrolled experimental fully matched patients who were followed for at least six months, and the ability to completely withdraw IS drugs by the end of one year without subsequent evidence of rejection. The primary endpoint of the study was to determine the percentage of patients off IS drugs for 2 years without evidence of rejection (89).

## Studies of Tolerance in HLA Mismatched Patients With Mixed Chimerism

### Feasibility Study of Tolerance Induction and Mixed Chimerism in Patients Given Combined HLA Mismatched Living Donor Kidney and Hematopoietic Cells Transplants Using TLI/ATG Conditioning Posttransplant

A feasibility study was performed at Stanford University to assess the safety and efficacy of infusing purified donor CD34+ hematopoietic progenitor cells into 4 patients given kidney transplants from HLA mismatched living donors in order to establish mixed chimerism using posttransplant TLI/ATG conditioning (90). Two patient pairs were unrelated and two were related haplotype matched patients, who were given a completely posttransplant regimen of 10 doses of 80cGy each and 5 doses of ATG during the first 10 days after transplantation (90).

Purified CD34+ cells (3-5x10^6 cells/kg) obtained from G-CSF mobilized donor peripheral blood were enriched on Miltenyi columns and infused into recipients shortly after the completion of TLI (90). Donor cells were harvested about 6 weeks before kidney transplantation and cryopreserved until thawing at the time of the cell infusion. T cell contamination of the purified CD34+ cells was below the threshold expected to cause GVHD (<1x10^5 cells/kg). Chimerism was measured using PCR based short tandem repeat analysis of purified subsets of blood cells. Maintenance IS drugs were prednisone and cyclosporine starting at standard of care doses. The goal of the study was to determine whether IS drugs could be withdrawn from patients with mixed chimerism by the end of the first year posttransplant.

Three of 4 patients developed transient mixed chimerism that was lost during the second and third months posttransplant, and none developed GVHD (90).

Immunosuppressive drug withdrawal was attempted in one patient who developed chimerism for about 1 month, and then donor specific unresponsiveness in the MLR after 7 months. IS drug withdrawal was completed at the end of one year without rejection episodes during withdrawal (90). However, the patient developed a rejection episode during the second year and was returned to standard of care maintenance therapy. Complete withdrawal was not attempted in the other two chimeric patients.

### Establishment of Transient Mixed Chimerism, Tolerance, and Complete IS Drug Withdrawal in Patients Given HLA Mismatched Living Donor Kidney and Hematopoietic Cell Transplants Using Anti-CD2 mAb, Thymic Radiation, and Cyclophosphamide Conditioning

A study of 10 patients given combined kidney and bone marrow transplants from HLA haplotype matched living donors was performed by investigators at Harvard University based on preclinical data generated in non-human primates and mini-swine (91–93). The preclinical studies showed that tolerance to MHC mismatched kidney transplants could be achieved using a conditioning regimen of TBI, thymic radiation and anti-T cell antibodies with transient chimerism for a few to several weeks (56, 71, 72, 94).

The follow up clinical study used cyclophosphamide instead of TBI for conditioning along with thymic radiation and anti-CD2 monoclonal antibodies. Maintenance IS drugs posttransplant were used for several months before complete discontinuation in 7 patients (91–93). Nine of 10 patients developed an “engraftment syndrome” with injury to the graft vasculature observed on biopsies obtained shortly after the loss of chimerism during the first month posttransplant (95). Nevertheless, 7 patients were subsequently withdrawn completely from IS drugs, and 4 of 7 remained off the drugs for 4 to 10 years without evidence of subsequent rejection (93). Graft loss was observed in 3 of 10 recipients who were not withdrawn from IS drugs during the first 3 years posttransplant, and 3 recipients developed acute and/or chronic rejection a few years after complete withdrawal (93).

The Harvard group examined mechanisms of immune suppression drug withdrawal and maintenance of graft function in their patients receiving HLA-mismatched combined kidney and bone marrow transplantation (CKBMT) that led to transient (< 3 weeks) donor cell chimerism (96, 97). They reported a marked increase in CD3+CD4+CD25highCD127lowFoxp3+ Tregs early after transplant and showed evidence that these cells originated from the thymic emigrants as well as peripheral expansion (96). Few Treg clones were identified as non-Tregs pre- transplantation which suggested that induction of Tregs from non-Tregs present prior to transplant was not a prominent contributor to the increased Treg population. High- throughput TCR sequencing from circulating cells enabled the tracking of clones pre and post-transplant, as well as the clones that infiltrated the kidneys on post transplantation renal biopsies (96). A relatively high proportion of clones in the post- transplant biopsies were also detected in the pre-transplant and post-transplant circulating CD4+ and CD8+ T cell populations. A relatively high percentage of clones in each biopsy of patients successfully weaned off immune suppression were identifiable as Tregs. These patients had a decrease of donor reactive conventional T cell clones identified in the pretransplant MLR such that non-donor reactive T cell clones represented the most abundant T cell clones in tolerant recipients (96, 97). In contrast, there was a relative high percent of donor reactive clones in the post-transplant biopsy of a graft rejector, and in patients who received conventional transplants (96, 97). The results suggested that peripheral immune regulatory mechanisms and clonal deletion reduce donor reactive clones. It is unclear if, and how, thymic irradiation and anti-CD2 monoclonal Ab administration promoted non-chimeric peripheral ‘tolerance’ in the Harvard protocol. It can be speculated that Tregs may be spared by treatment with siplizumab and thymic irradiation, and that treatment may result in immune modulatory responses that enhance Treg emigrants from the thymus, as well as their peripheral expansion.

### Establishment of Persistent Mixed Chimerism and Partial IS Drug Withdrawal During the First Year Posttransplant in Patients Given HLA Mismatched Living Donor Kidney and Hematopoietic Cell Transplants Using TLI/ATG Conditioning

Based on the results of the HLA matched tolerance induction trial using TLI/ATG conditioning, Stanford investigators performed a follow up trial to determine whether immune tolerance and complete IS drug withdrawal could be achieved in 22 recipients of HLA haplotype matched living donor kidney transplants (87, 88).The study had 3 goals:1) Establish persistent mixed chimerism for at least one year, 2) During the first year reduce IS drugs to tacrolimus monotherapy in persistent chimeras, and 3) Determine whether tacrolimus monotherapy could be discontinues during the second year.

Recipients were given 10 doses of 120cGy each of TLI and 5 doses of ATG as in the fully matched study, and an infusion of donor CD34+ cells and a defined dose of T cells that was escalated from 3x106 cells/Kg to 150x106 cells/Kg in order to facilitate mixed chimerism (87). During the study the CD34+ dose was increased to a minimum of at least 10x106 cells/kg, by adding one dose of the mobilizing agent, plerixafor, to 5 doses of G-CSF administered to donors. Recipients were given maintenance IS drugs during the first year starting with prednisone, MMF, and tacrolimus. The MMF and prednisone were withdrawn, and patients were maintained on tacrolimus monotherapy at the end of one year, if they had persistent mixed chimerism by STR analysis, no evidence of rejection on protocol biopsy, and no evidence of GVHD (87, 88). Ten of the 22 patients developed chimerism for at least one year while reducing three IS drugs to monotherapy. Thus, the first two goals were achieved.

### Study of Withdrawal of Tacrolimus Monotherapy in Mixed Chimeras During the Second Year Posttransplant in Patients Given HLA Mismatched Living Donor Kidney and Hematopoietic Cell Transplants Using TLI/ATG Conditioning

In order to achieve the third goal, complete IS drug withdrawal was attempted during the second year in the 10 haplotype matched patients with persistent chimerism at the end of one year on tacrolimus monotherapy (87, 88). Tacrolimus dosing was gradually tapered while monitoring the chimerism levels of whole blood and T cells, as well as serum creatine values monthly. Levels of T cell chimerism in all 10 patients at the end of the first year, and before tacrolimus tapering, were below 20% (mean 10%) (88).

Tapering to subtherapeutic levels or to discontinuation of tacrolimus was associated with loss of chimerism in the first 6 patients (88). Three of the 6 developed mild acute rejection episodes that were resolved with standard of care treatment. The latter patients as well as 3 of 6 who lost chimerism without evidence of rejection were returned to maintenance IS drugs (88). The remaining 4 of 10 patients were maintained on therapeutic levels of tacrolimus monotherapy with persistence of mixed chimerism with up to 5 years of observation. One of the latter patients with proteinuria, and a biopsy that showed glomerulonephritis without rejection was switched to MMF monotherapy. Graft function has remained normal in these patients.

The instability of mixed chimerism and dependence of chimerism on continuation of IS drugs after one year in these patients contrasts with the observations of mixed chimerism in MHC mismatched rodents that were conditioned with TLI/ATG and given combined organ and bone marrow transplants (38, 39, 44, 75). Mixed chimerism observed starting one month after the donor bone marrow infusion was uniformly stable in the absence of IS drugs even with T cell chimerism levels below 20% in the majority of recipients (38, 39, 44, 75). Stability of mixed chimerism was observed in some groups of rodent recipients given a brief course of cyclosporine, and subsequent withdrawal of cyclosporine (61).

The observation of IS drug dependent mixed chimerism in the patients with HLA mismatched kidney transplants, and the IS drug independent mixed chimerism in MHC mismatched rodents using the same TLI/ATG conditioning regimen suggests that the rodent and human immune systems differ in their responses to the combined transplants. The instability of mixed chimerism in studies of patients given hematopoietic cell transplants to treat hematologic malignancies and anemias based on genetic mutations is consistent with the observations observed in the kidney transplant patients.

It is possible that the low levels of T cells chimerism (<20%) observed in the haplotype matched kidney transplant patients at the end of the first year contributed to the instability of chimerism during IS drug tapering during the second year, and that increased levels of T cell chimerism will allow for complete withdrawal of IS drugs without subsequent rejection. Investigators at Stanford are currently intensifying the conditioning regimen to increase the levels of T cell chimerism to determine the impact on the stability of chimerism during taper and discontinuation of IS drugs during the second year posttransplant.

## Studies of Organ Transplant Acceptance in HLA Mismatched Patients With Complete Chimerism

### Establishment of Complete Chimerism and Withdrawal of IS Drugs in Patients Given HLA Mismatched Living Donor Kidney and Hematopoietic Cell Transplants Using Pre and Posttransplant Conditioning With TBI, Fludarabine, and Cyclophosphamide

Investigators at Northwestern University used a conditioning regimen for their studies of kidney and hematopoietic progenitor transplantation that was developed at Johns Hopkins University for treatment of hematologic malignancies with HLA mismatched bone marrow transplantation (27). The conditioning regimen included administration of pre-and post-transplantation cyclophosphamide (PTCy), a well- established potent inhibitor of HVG and GVH reactions. More than 50 years ago it was demonstrated that cyclophosphamide was effective at delaying the rejection of MHC mismatched mouse skin allografts when given after compared to before the graft (98). Durable MHC mismatched mouse skin allografts however, were attained only when the combination of pretransplant host conditioning was followed by an infusion of ≥50 million allogeneic donor spleen cells and 48-72 hours thereafter by an intraperitoneal injection of high dose cyclophosphamide (33).

The use of PTCy was translated to a clinical protocol in cancer patients receiving HLA haploidentical bone marrow in a two-cohort study intended to determine if cyclophosphamide could improve engraftment (99) by mitigating HVG reactions. In cohort 1, patients were conditioned with fludarabine 30 mg/m 2 on days −6 to −2, TBI 2 Gy on day −1, and PTCy 50 mg/kg on day + 3. Patients received post grafting immune suppression with mycophenolate mofetil and tacrolimus. In cohort 2, patients received the same regimen, plus the addition of pre-transplantation cyclophosphamide. The majority of patients in the second cohort achieved full donor chimerism. In 2008, a two- center study established PTCy as an acceptable platform for GVHD prophylaxis after haplo-matched BMT (100). Patients received intermediate intensity fludarabine/Cy/TBI pretransplant host conditioning with PTCy on days +3 and +4. The majority of patients achieved full donor chimerism, the incidence of all grades of acute GVHD was 34% and that of severe grade III/IV acute GVHD was 6%. The incidence of chronic GVHD was < 25% (100).

The main mechanism by which PTCy mitigates bi-directional alloreactivity to reduce the incidences of graft rejection and GVHD is to eliminate donor reactive intrathymic host T cells and post thymic host T cells that cause graft rejection, and to eliminate the proliferating alloreactive donor T-cells necessary for GVHD (101, 102). Post transplantation cyclophosphamide was reported to spare *foxp3* + regulatory T cells (T regs), possibly due to the high expression of aldehyde dehydrogenase, an enzyme that metabolizes cyclophosphamide (103).

In the Northwestern protocol, donor cells injected into conditioned recipients included CD34+ cells, and α,β T cells and a unique population of facilitator cells (FC). Two major CD8+ FC subpopulations were described, one is CD56dim/− and the other CD56bright. The majority of CD56dim/− FC were also positive for CD3ϵ and HLA-DR and negative for the dendritic cell markers CD11c and CD123. The CD56dim/− FC comprised approximately half of the FC total. The majority of the CD56bright FC subpopulation were CD19+, CD11c+, CD11b+ and CD3ϵ− and comprised just under half of the FC total (104).

The intensity of host conditioning combined with the composition of cells in the donor inoculum resulted in kidney transplant patients with early and complete conversion to donor chimerism in most recipients (104–106). Complete chimerism is not tolerance to donor alloantigens by recipient immune cells, since no recipient immune cells remain after the HCT procedure. As noted above, non-responsiveness to donor antigens in complete chimeras is due to self-tolerance. Rather, the issue in complete chimeras is GVH alloreactivity and its complications of GVHD.

It is difficult to assess the contribution of the unique FC population in promoting conversion to complete chimerism and mitigating GVH alloreactivity. In single and multi- center studies of cancer patients that received haploidentical bone marrow transplants using unmanipulated donor cell infusions with pre- and post-transplantation cyclophosphamide, over 90% of recipients attained complete donor chimerism (107, 108). The cumulative incidence of 100-day grade 3-4 acute GVHD, and immune suppression requiring chronic GVHD was <8% and 20%, respectively (107, 108).

The Northwestern group reported at least one year of follow-up in 37 patients (36 at Northwestern, 1 at Duke University) treated using their protocol (104–106). Recipients were initially maintained on tacrolimus and mycophenolate-based immune suppression. At six months, if stable renal function, and a normal protocol biopsy were noted, then mycophenolate was discontinued in the chimeric patients. Testing for the absence of donor specific antibodies in complete chimeras would not offer information about the potential for allograft rejection as the recipient is all donor type. Tacrolimus was weaned over the six months that followed, and fully withdrawn at one year if patients continued with complete donor chimerism and normal renal function.

Among the 37 patients, 26 achieved full donor chimerism and 23 (62%) were removed from immune suppression medication. Eight patients with transient chimerism required immune suppression. Two patients failed to establish donor cell chimerism at any level. Two patients had kidney allograft loss during the first year after transplant, and two other patients with full donor chimerism developed immune suppression dependent GVHD one of who died from GVHD (104–106).

### Summary of Outcomes of the Clinical Tolerance Protocols With Combined Kidney and Hematopoietic Cell Transplantation

The Harvard, Northwestern, and Stanford University protocols vastly differ from one another albeit all use the concept of combining donor hematopoietic cell infusions, host transplant conditioning, and planned immune suppression drug withdrawal from kidney transplant patients. Comparisons of pros and cons are difficult especially given that relatively few patients have been transplanted therefore the accuracy concerning toxicity, complications and outcomes remains somewhat speculative (**Table 1**).

**Table 1:** Comparison of Relative Safety of Clinical Tolerance Protocols.

	Northwestern	Boston	Stanford
Intensity of Conditioning*	+2.5, traditional BMT protocol	+1, nonmyeloablative	0, lowest intensity nonmyeloablative
Conditioning Regimen**	Flu-Cy/TBI and PTCy	Cy/TR/Anti-CD-2/Rituxan	TLI-ATG
***Grade 4 neutropenia	100%	100%	< 5%
***Grade 4 thrombocytopenia	100%	100%	< 5%
***Grade 3 Anemia	100%	Not reported	<1%
**ICU stay during 1^st^ 90 days**	Estimated > 5%	Unclear	None
**Chimerism goal**	Complete	Transient	Persistent mixed
**GVHD risk**	>10%	No	No
**GVHD related death**	Yes	No	No
**** Apparent need for medical teams to support post-transplant care beyond SoC renal transplant team	YES: BMT unit, ICU, Pulmonary, Urology, Infectious Diseases, Gastroenterology	YES: BMT unit	No
**Ability to directly translate to recipients of deceased donor transplants**	No	No	Yes

*Transplant conditioning intensity score based on Ref (109).

**Flu, Fludarabine; Cy, cyclophosphamide; TBI, total body irradiation; PTCy, post-transplant Cytoxan; TR, thymic irradiation; TLI, total lymphoid irradiation; ATG, anti-thymocyte globulin.

***Based on Common Terminology Criteria for Adverse Events (CTCAE; Version 5. https://ctep.cancer.gov/protocoldevelopment/electronic_applications/docs/ctcae_v5_quick_reference_5x7.pdf).

****SOC, standard of care.

The Northwestern approach was akin to traditional allogeneic HCT for hematologic malignancies with predictable and significant toxicities, some life threatening, and attainment of complete donor chimerism. Immune suppression drug withdrawal in the setting of 100% donor chimerism does not reflect HvG tolerance, and a concern with this approach is GVH alloreactivity and development of GVHD in some patients. GVHD was not observed in the Harvard and Stanford protocols.

The Stanford protocol was well tolerated and associated with few if any toxicities above and beyond a standard of care transplant. The conceptual framework is predicated on the protection afforded by persistent mixed chimerism that prevents bi- directional HvG and GvH reactions. Immune suppression independent persistent mixed chimerism in HLA mismatched transplants was not reliably been achieved (87, 88). Rather drug minimization with the use of low dose monotherapy was reported to be required for persistence of chimerism (88). The Stanford protocol is adaptable to deceased donors (https://clinicaltrials.gov/ct2/show/NCT04571203) as host conditioning is entirely post kidney transplant.

The Harvard protocol was associated with the side effect of “engraftment syndrome” in 9 of 10 patients in the first month posttransplant (95). The syndrome was a manifestation of vascular injury to the kidney graft shortly after the loss of chimerism (95). The syndrome was not observed in the Northwestern study, nor in the Stanford study of fully HLA matched patients, but was observed in 2 of 22 HLA mismatched patients shortly after the loss of chimerism in the Stanford study (88). Whereas kidney graft loss was observed in the first three years of Northwestern and Harvard studies, it was not observed in the Stanford study (88, 95). The Harvard study focused attention on mechanisms of peripheral tolerance, the role of Tregs, and acquired deletion of donor reactive recipient T cells in patients with transient chimerism who were off drugs (96).

The Stanford study showed evidence of donor specific unresponsiveness in HLA matched and mismatched chimeric recipients, but did not assay for clonal deletion (85–88). The Northwestern study did not assay recipient cell immune responses due the development of complete chimerism.

## Comparisons of Protocols Used to Treat Patients With Hematologic Malignancies Versus Protocols to Induce Tolerance in Kidney Transplant Patients

### Comparison of Intensity of Host Conditioning for HCT Treatment of Hematologic Malignancy Versus Tolerance Induction

That host conditioning pre-hematopoietic cell infusion is essential for the engraftment of donor cells is beyond reproach. There are dozens of published host conditioning regimens that enable donor cell engraftment, and they vary significantly in the chemotherapy and radiation components included, and in their intensity profile.

Defining the intensity of the conditioning regimen is based on the type and dose of chemotherapy and/or radiation administered, the expected duration and severity of pancytopenia (irreversible, prolonged, minimal), and the requirement for stem cell support (essential, required, optional) (109, 110).

It is well established in allogeneic HCT cancer recipients that as the intensity of host chemo-radiation conditioning increases, donor hematopoietic cell engraftment is more easily attained and conversion to complete donor cell chimerism is the result (110, 111). Complete chimerism (>95% donor type) is a desired goal in cancer patients because complete chimerism by definition does not create tolerance, and donor cell tolerance is not advantageous to patients with cancer (112). Rather, post thymic alloreactive donor T cells that accompany the infused donor cell inoculum are required to provide beneficial anti-tumor reactions important in mediating cancer cures. Complete chimerism, however, comes with the risk of acute and chronic GVHD which is mediated by alloreactive donor immune cells to the target tissues of GVHD (111). The consequences of GVHD are the main limitation to safe allogeneic transplantation. To use common transplant parlance, the ‘holy grail’ so to speak in allogeneic HCT for cancer patients is to separate the deleterious graft versus host reactions from the beneficial graft versus tumor reactions.

Numerous trials in cancer patients compared clinical outcomes that helped to define conditioning dose intensity. During the initial development of reduced intensity conditioning regimens for cancer patients 2 Gy of TBI was compared to 2Gy TBI combined with 90 mg/m2 fludarabine (Flu) (112). Pretransplant conditioning with 2Gy TBI alone resulted in a predictable 10-14 days of marked cytopenia, yet 20% of the patients recovered endogenous hematopoietic cells that led to donor hematopoietic graft rejection. In order to reduce the high donor cell rejection rate, Flu was added to the 2 Gy TBI, which resulted in a significant decrease in donor hematopoietic rejection to 3%. The median donor T-cell chimerism levels were significantly higher in the TBI-Flu arm compared to the TBI arm at day +28 (90% vs. 61%, p <0.001) owing to the more profound host immune cell depletion with the addition of Flu that facilitated donor cell engraftment. Patients without disease relapse in the TBI-Flu arm converted to complete donor cell chimerism by day +90. For patients on the TBI-Flu arm, the incidence of clinically significant acute and chronic GVHD was 46% and 48%, respectively, and the transplant related mortality at 1 year was 5%. This randomized trial demonstrated the importance of fludarabine in augmenting the ensuring prompt and durable conversion to complete donor cell chimerism (112).

As the intensity of host conditioning increases, the likelihood of developing clinically significant Grades 3, 4 and 5 adverse event (AE) toxicities increase (109, 110). These toxicities include but are not limited to the GI tract (mucositis/colitis/typhlitis), lungs (pneumonitis and diffuse alveolar hemorrhage), heart (chemotherapy induced cardiac dysfunction and radiation induced vascular damage), and liver (sinusoidal obstructive syndrome). With increases in conditioning intensity marked and prolonged cytopenia develops which may lead to severe neutropenic infections, sepsis, as well as the need for red cell and platelet transfusion support. The regimen related tissue toxicities typically resolve within the first eight weeks of the cell infusion yet they are associated with unanticipated hospitalizations, significant patient morbidity, the need for consultation with medical specialty (such as BMT physicians, pulmonary, ICU, gastroenterology, infectious diseases) teams, and increased transplant related mortality.

A transplant conditioning intensity (TCI) scoring system was developed to help investigators standardize nomenclature and allow a comparison of the many differing regimens (110). For example, and with particular relevance to kidney tolerance host conditioning regimens, points would be assigned based on the doses of TBI, Flu, and Cyclophosphamide (110). The higher the TCI score the more intense the regimen. The performance of the TCI score was tested in over 8200 BMT recipients and regimens were grouped as low intensity (score of 1 and 2), intermediate intensity (score of 2.5- 3.5) and regimens with scores of >3.5 were considered high intensity (110).

Intermediate score regimens were highly predictive of increased early (day +100 and +180) regimen related toxicity and mortality compared to low intensity regimens.

The MGH tolerance regimen in their patients without malignancy consisted of 60 mg/kg of cyclophosphamide on days −5 and −4 with respect to transplantation; a humanized anti-CD2 monoclonal antibody (MEDI 507, MedImmune) on days −1, 0, and +1, thymic irradiation (700 cGy) on day −1, +/- rituximab 375 mg/m2 days −7 and −2 (91). In an effort to mitigate the toxicities of the high-dose cyclophosphamide including the severe cytopenia, gastrointestinal side effects and cardiotoxicity, and to help eliminate the “engraftment syndrome” that occurred in 9 of 10 patients the conditioning regimen was changed and total body irradiation (1.5 Gy x 2, total dose of 3 Gy) was substituted for cyclophosphamide (113). The MGH regimens would score as low intensity conditioning with one point assigned for cyclophosphamide, or 3 Gy TBI. Marked cytopenia would be of short duration and the need for a donor stem rescue would be desirable but not essential or required.

The Northwestern kidney tolerance regimen consists of cyclophosphamide 50mg/kg on days -3 and +3, 2 Gy TBI on day -1, and Fludarabine total dose of 90mg/kg divided equally on days −5, −4, −3 (104, 106) is intermediate intensity with a score of 2.5 based on 1 point for TBI dose, 0.5 points for fludarabine, and 1 point for cyclophosphamide.

This degree of intensity is associated with severe neutropenia, the need for G-CSF administration in all recipients, and the requirement for transfusion support. A “rescue” donor cell graft is not essential yet is highly desirable (required) in order to avoid prolonged blood count recovery and the associated significant health risks.

The Stanford conditioning regimen that uses TLI-ATG falls below the TCI scoring system because the combination of TLI and ATG does not induce clinically significant cytopenia or regimen related organ (gastrointestinal, pulmonary or liver) toxicity. There is no need for transfusion support. The unexpected 100-day re-hospitalization rate in over 600 cancer patients transplanted from HLA matched and mismatched related and unrelated donors was 25% compared to over 80% for patients that were transplanted using 2 Gy of TBI or 2 Gy TBI-Flu (114). The 1-year re-hospitalization rate in the 38 recipients of living related HLA matched and mismatched donor kidneys using the Stanford tolerance regimen was 13%, a value that is not different than contemporaneously treated kidney transplant recipients at the same institution using standard of care methods (87, 88).

The assessment of the risk of developing clinically significant AEs and mortality resulting from toxicity of the intensity of host conditioning is based on scores and indices specifically developed to measure the conditioning regimen intensity. Whereas the MGH and Stanford protocols are non-myeloablative, low intensity regimens the Northwestern protocol follows a traditional BMT reduced intensity conditioning (RIC) regimen of intermediate intensity and that is associated with increased regimen related morbidity and mortality (110).

### Comparison of Donor Cell Inoculum in HCT Treatment of Hematologic Malignancy Versus Tolerance Induction

The composition of the donor cell inoculum is also an important determinant in whether engraftment and chimerism is transient, persistent and mixed, or results in conversion to complete door type. The cellular composition contributes to the risks of developing acute and chronic GVHD, and post-transplant infections, and the risk of transplant related mortality (115–119). Despite over 50 years of allogeneic HCT, however, the immune and progenitor cell composition of grafts is typically not well characterized in clinical practice, except for the number of total nucleated cells (TNC), and CD34+ and CD3+ cells. There is wide variation in the cellular composition even when factors like donor age, sex, the method of collection, and source of hematopoietic cells (marrow or mobilized blood) is controlled (120). The time of day of collection can influence the donor cell composition; HSCs and other progenitor populations do not steadily or randomly circulate under homeostasis, but rather follow a physiologically regulated, rhythmic circadian oscillation of release (121). A report of 85 healthy donors confirmed that afternoon apheresis collections, when the level of circulating catecholamines are at their lowest, resulted in significantly higher average CD34+ cell yields compared to products in which donors underwent apheresis in the morning (122).

As a general consideration, donor cell engraftment is facilitated by increasing the number of donor TNC (total nucleated cells), and CD34+ and CD3+ T cells infused. The cellular composition should also be considered in combination with the intensity of host conditioning. For example, as the intensity of host conditioning is reduced higher numbers of TNC, CD34+ and CD3+ T cells were required to support donor cell chimerism (116, 123, 124). Using low intensity TLI-ATG conditioning in cancer patients who received unmanipulated G-mobilized apheresis products that contained roughly 200-400 x106 CD3+ T cells/kg and >5 x106 CD34+ cells/kg only 60% of recipients converted to complete chimerism by +90 days after the cell infusion (26, 114). Even more challenging is the combination of a low intensity conditioning regimen with a low number of CD3+ T cells, and in this scenario mega-doses (>10 x106/kg) of CD34+ cells were important in helping establish engraftment (125–127). In a small series of cancer patients that received low intensity 2 Gy TBI conditioning, followed by the infusion of a graft containing a low number of column enriched CD34+ cells (range of 3-5 × 106/kg) combined with 2 ×106/kg CD3+ cells, 4 of 5 patients had low levels of transient chimerism that was not sustained beyond three months (127). Taken together, these data confirm an important relationship between host conditioning intensity and the cellular composition of the donor inoculum in terms of achieving chimerism.

In assessing the MGH protocol for patients without malignancy the graft source and cell doses were not specified. Based on the body of their work, however, it is reasonable to presume unmanipulated donor bone marrow harvest products were obtained at the time of kidney harvest. Decades of clinical bone marrow transplantation has confirmed that the interquartile (equal to the difference between the 75th and 25th percentiles) range for TNC and CD34+ cells in a bone marrow graft is about 1.5-4.0 x108/kg and 2-4.5 x106/kg, respectively, and CD3+ T cells range between 20-40 x106/kg for (128). When bone marrow harvest cell doses are combined with the low intensity MGH conditioning regimen, and across mismatched HLA barriers, it is not surprising that donor cells were detectable for <21 days post transplantation. A potential limitation to using bone marrow as a graft source is the low number of hematopoietic cells that can be obtained. In contrast, cell products collected by apheresis following a variety of donor mobilization strategies allows far greater numbers of TNC, and CD34+ and CD3+ T cells that can be, or not, manipulated or enriched.

The Northwestern protocol uses a unique donor cell composition that is a challenge for the reader to understand because details are considered proprietary to a commercial entity and was based on preclinical murine models. The publications highlight that using the CliniMACS (Miltenyi Biotec) system mature GVHD-producing and antigen- presenting cells are removed while HSC, FC, and progenitor cells are retained. The dose of CD34+ cells range from about 1-16 x106/kg, the dose of α,β T cells appear set at 3.8 x106/kg and FC range from 2-12 x106/kg (104–106). It is unclear if the α,β T cells represent an enrichment population with few contaminating cells, or simply a volume adjusted fraction of the CD34+ flow through. Nonetheless, the doses of cells combined with intermediate intensity host conditioning is sufficient to result in conversion to complete donor cell chimerism in the majority of recipients.

There is a panoply of cells in the donor inoculum that appear important in promoting engraftment, and that are not associated with inducing GVHD. In this regard, there is abundant preclinical literature to support that Tregs of donor and recipient origin promote donor hematopoietic engraftment or enable persistent mixed chimerism without inducing GVHD (48, 129, 130). The analysis of 32 cancer patients that received allogeneic HCT and achieved persistent mixed instead of complete chimerism showed that the proportion of Treg cells in the circulation was increased in patients with mixed chimerism (131). The Treg cells were comprised of equal numbers of donor and host-derived regulatory cells. The dendritic cells in the patients with mixed chimerism had a tolerogenic programmed death ligand-1 (PD-L1) profile. The T cells from patients with mixed chimerism showed reduced cytotoxicity against host target cells *in vitro* that was restored following depletion of CD4+ Treg cells. The aggregate of data supports the contention that suppression of bi-directional alloimmune responses in mixed chimerism may be enhanced through peripheral cell-based regulation and raises the potential therapeutic options of Treg cells (131). This concept is currently being investigated in a Stanford and Northwestern collaborative phase 1 study (https://clinicaltrials.gov/ct2/show/NCT03943238) using the Stanford host TLI-ATG conditioning regimen, a donor graft enriched for CD34+ cells combined with a defined CD3+ T cell dose and a recipient Treg cell infusion in HLA mismatched living donor kidney transplant recipients.

### Comparison of Post-Transplant Immune Suppressive Antibodies and Drugs for HCT Treatment of Hematologic Malignancies Versus Tolerance Induction

The addition of donor derived hematopoietic cells imparts new immunologic phenomena not previously considered in clinical organ transplantation with a crossing of a double barrier, there is the known HVG reactions yet this is combined with GVH reactions. Traditionally, high intensity myeloablative and immune suppressive host conditioning regimens administered before the donor cell infusion were to eliminate HVG reactions and facilitate donor cell engraftment, while the need for post-infusion immune suppression mitigated the complications of GVH reactions. With the development of lower intensity regimens in which residual host hematopoietic and immune cell compartments persist, the importance of post grafting immune suppression focused attention to the need to mitigate HVG reactions. The Seattle group used DLA- identical littermate dogs given donor bone marrow cell infusions at clinically relevant doses of 1.9 to 4.4 × 108 TNC/kg within 4 hours of completing low intensity 2 Gy TBI (132). Recipient animals received post grafting immune suppression with cyclosporin (CSP) alone, CSP combined with methotrexate, or CSP combined with mycophenolate mofetil (MMF). Dogs that received post grafting immune suppression with the combination of CSP and MMF had sustained mixed chimerism whereas dogs in the other groups had no, or a low level of transient chimerism that did not persist beyond 30 days. Reducing the dose of TBI to 1 Gy combined with post grafting CSP and MMF failed to result in donor cell chimerism. This important contribution confirmed the need of pharmacological immune suppression post grafting to induce mixed chimerism and inhibit HVG reactions.

The Northwestern and Stanford protocols albeit very different from one another, use as a backbone post grafting Tacrolimus combined with MMF, in part because the above article so heavily influenced, in the right direction, the field (85–88, 104–106). Weaning of immune suppression is dependent on clinical outcomes with a planned tapered to cessation within 12-18 months of organ transplantation. The MGH protocol used single agent CSP as a post grafting immune suppression (91–93).

There are additional post grafting immune suppression considerations. The MGH team used pre- and post-grafting anti-CD2 humanized monoclonal antibody (MEDI- 507), siplizumab, in their ten patient published series (91–93). CD2 is a non-essential cell adhesion molecule found on the surface of T cells and natural killer (NK) cells that interacts with lymphocyte function-associated antigen-3 (LFA-3/CD58) on APCs, most commonly macrophages (133). Yet CD2 has additional multifunctionality acting as a co- stimulatory signal on T and NK cells, and is upregulated on memory and activated T cells. Anti-CD2 monoclonal Abs have been shown *in vitro*, and in clinical studies to induce immune modulatory effects that include upregulation of Treg cells (134). The specific mechanism(s) by which anti-CD2 Ab treatment contributed to the ability to wean immune suppression medication remains to be more clearly defined.

The Northwestern group used high-dose, post-transplantation cyclophosphamide (PTxCy). Post-transplantation cyclophosphamide represents the culmination of over 50 years of preclinical research dedicated to inducing immunologic tolerance (27). The use of PTxCy is particularly attractive because the treatment is inexpensive, strikingly effective at depleting alloreactive HVG and GVH T cells, and requires no special manipulation beyond the administration of high dose IV chemotherapy. Post grafting anti-CD2 Ab administration and PTxCy have important roles in contributing to the clinical outcomes of the kidney tolerance protocols.

### Approaches to Prevent GVHD After HCT Treatment in Hematologic Malignancies and Tolerance Induction

A main limitation to safe treatment of hematologic malignancy with allogeneic HCT is that donor T cells infused at the time of transplant are the critical mediators of GVHD even when GVHD appears months after the infusion. The intensity of alloreactivity is proportional to the degree of HLA mismatch between donor and recipient (24). Studies that infused a T cell depleted (TCD) donor graft (CD3+ T cells < 105/kg) following high intensity myeloablative host conditioning reported significantly less acute and chronic GVHD (24). Post grafting immune suppression was not needed in these trials because the high intensity conditioning effectively eliminated host immunity and prevented for the most part HVG reactions and graft rejection. The infusion of a product that contained >3-log fold *in vitro* T cell depletion sufficiently removed enough alloreactive T cells that GVHD was markedly reduced (135). Yet despite the marked reduction in GVHD, overall patient survival was not increased in these studies due to a higher rate of disease relapse (there is reduced GVT reactions without sufficient number of donor T cells), and increased patient mortality due to infection. T cell replete syngeneic twin transplants developed little GVHD even in the absence of post-transplant immune suppression medication (136). The infusion of donor lymphocytes (DLI) to convert mixed to complete chimerism, or as treatment for disease relapse induced acute GVHD in a dose dependent manner (137). GVHD was not observed when the dose of CD3+ T cells in an unmanipulated DLI was <10 x 106/kg, whereas 10%, 30% and 50% of recipients experienced clinically significant and severe GVHD when infused with a DLI containing T cells doses of 10 x106 CD3+ T cell/kg, 50 x106/kg and 100 x 106/kg, respectively (137).

The risk of GVHD extends beyond just the dose of infused T cells and is also dependent on the level of chimerism attained. Whereas mixed chimerism protected against GVHD, complete chimerism associated with the risk of developing GVHD. Studies confirmed that there is a precipitous fall in the risk of GVHD once there is more than 10% residual lymphoid (CD3+) cells (138), and virtually no GVHD risk if the percent donor T cells remained below 75%. The majority of patients treated using the Northwestern protocol rapidly converted to complete donor type and therefore patients on this protocol were at risk for acute and chronic GVHD. In fact, GVHD has been reported by the Northwestern investigators including death from GVHD and its associated complications.

The removal of Tnaive cells from the donor cell inoculum by CD45RA column depletion is a platform to promote donor cell engraftment with a low risk of clinically significant GVHD even after conversion to complete donor chimerism (139, 140).

Enrichment for CD8+ T memory cells following CD45RA depletion also promoted conversion to complete chimerism with perhaps even lower GVHD risk then a CD45RA depleted graft as CD4+ memory T cells may promote GVHD (141, 142). Another graft manipulation strategy that may not significantly affect the likelihood of donor cell engraftment yet that limits GVH reactivity and that is in clinical trials is based on depletion of the lymphocyte population primarily responsible for GVHD, namely, T lymphocytes carrying the αβ chains of the T-cell receptor (TCR), coupled with B-cell depletion accomplished through the use of an anti-CD19 monoclonal Ab (143). These studies confirmed prompt donor cell engraftment with a low risk of GVHD even across major HLA barriers (143). Clinically significant viral infections limit the broad applicability of this strategy. The point being that there are a multitude of cellular components in the donor inoculum that may have significant impact on the outcome. It would be helpful to clinical researchers in the field if the three tolerance programs invested effort to more comprehensively identify the cellular composition of the donor graft used in their trials and this would include providing the details regarding populations of cells with unusual phenotypes.

## Realtionship Between Chimerism and Tolerance in Laboratory Animals and Humans

### Differences in the Stability and Linkage of Chimerism to Tolerance in Humans Versus Laboratory Animals

The rodent models summarized in this review uniformly demonstrated that mixed chimerism and tolerance were stable after MHC matched and mismatched combined hematopoietic progenitor and organ transplantation (38, 39, 44–52). The observations in the cited rodent models were not reflected in large animal models and in humans. However, the use of additional strains of rodents and additional conditioning regimens may provide improved models for large animals and humans. Mixed chimerism was frequently transient, unstable, and dependent on the presence of IS drugs in humans (87, 88). The strong link between tolerance and persistent chimerism in rodents was also not observed in humans. Acceptance of organ grafts and tolerance of recipient immune cells to donor alloantigens continued in most MHC matched and in some mismatched humans withdrawn from IS drugs even after loss of mixed chimerism (85–88).

In the case of complete chimerism, concordant observations were made in humans and laboratory animals. Complete chimerism was achieved in HLA mismatched patients given combined kidney and hematopoietic progenitor transplants at Northwestern University, and was stable (104–106). Acceptance of donor organ grafts in the complete chimeric patients was based on self-tolerance rather than on tolerance of recipient immune cells to alloantigens.

The tolerance induction clinical trials performed at Stanford and Harvard Universities represented a bench-to-bedside translation of preclinical models of mixed chimerism and organ transplantation tolerance to clinical medicine to establish proof of principle that scientific approaches can be used to treat diseases in humans. Each used a center-specific approach to establish mixed chimerism: In one case there is transient, short duration mixed chimerism, in another persistent and often permanent mixed chimerism. There are pros and cons with each approach and when considered as an aggregate they provide insight into some of the fundamental concepts and mechanisms that underlie immune suppression drug withdrawal chimerism-based tolerance protocols.

Mixed chimerism in these studies was uniformly above 1% of donor type cells in the recipient blood and lymphoid tissues, and in almost all instances above 10%. This level of chimerism is referred to as “macrochimerism”. In contrast, some studies of organ with or without hematopoietic progenitor transplantation have reported the development of stable “micro-chimerism”. In 1993 Starzl and colleagues reported a small cohort of liver transplant recipients that spontaneously developed very low levels of donor hematopoietic cell chimerism detectable only by high resolution PCR testing that was subsequently termed micro-chimerism (<1% donor type: the liver itself can act as a hematopoietic cell reservoir and bring forth donor hematopoietic progenitor cells after transplantation). Subsequent withdrawal of immune suppression medication with maintenance of normal graft function was seldom accomplished (144, 145). This report led to the development of protocols that evaluated combined deceased donor organ and vertebral body (VB) bone marrow cell infusions to induce chimerism and promote drug minimization and graft survival. By 2003, it was reported that 400 liver, 125 kidney, 28 heart, and 25 kidney and pancreas transplants received VB bone marrow cell infusions from their deceased organ donor (144–149). Yet unlike the mixed chimerism and transplant tolerance approaches used above for HLA mismatched recipients, host conditioning was not given in these early trials and consequently anything beyond transient micro-chimerism was not established.

To provide context to chimerism-based kidney tolerance protocols the lessons learned from more than five decades of clinical allogeneic HCT in more than 1 million cancer patients that received donor grafts should be considered. Patients receiving allogeneic hematopoietic cells are prepared with pre-hematopoietic cell infusion ‘host conditioning’ that consists of chemotherapy alone or in combination with radiation therapy and/or antibodies directed to immune cells. The pretransplant host conditioning is essential to establish donor hematopoietic cell chimerism and serves two critical purposes: to deplete and suppress the recipient immune cells that would otherwise reject the infused donor cells, and to deplete host bone marrow progenitor cells from their marrow niches to create ‘marrow space’ and enable donor stem cell replacement and engraftment.

### Hypothesized Relationships Between Chimerism and Tolerance in Humans and Laboratory Animals

**Figure 4:**
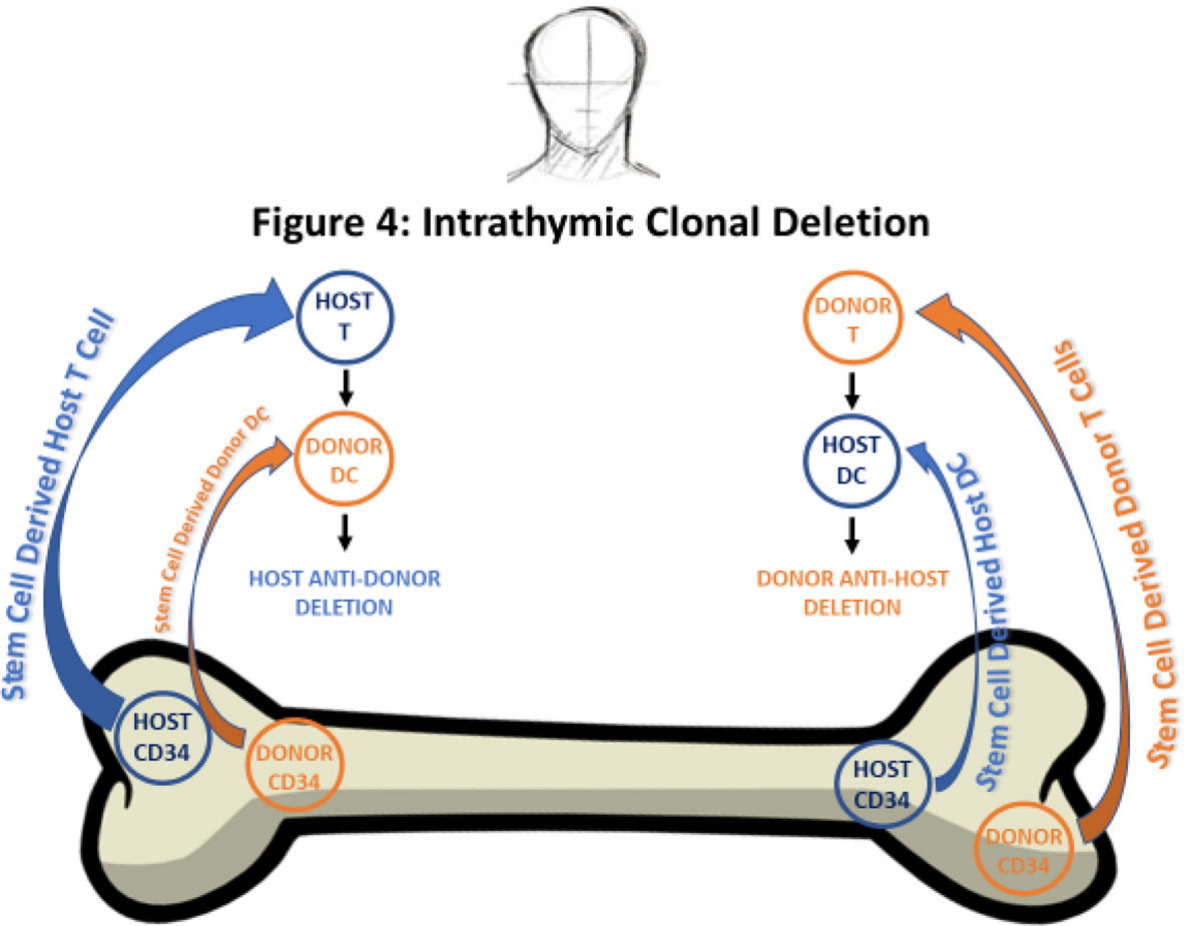
Intrathymic Clonal Deletion: Newly generated donor and host T cells do not cause GVHD or graft rejection in mixed chimeras. Newly generated naïve donor T cells are clonally deleted against host alloantigens when T cell precursors interact with donor intrathymic DCs derived from residual host CD34 cells. Newly generated naïve recipient T cells are clonally deleted when T cell precursors interact with intrathymic DCs derived from injected donor CD34 cells.

In context of protocols that use donor hematopoietic cells, tolerance is perhaps easiest to conceptualize in the setting of persistent mixed chimerism. With persistent mixed chimerism, donor APCs in the thymus present donor Ag to developing host T cells and induce deletion of alloreactive HVG T cells. Similarly, with mixed chimerism host APCs in the thymus present host self Ag to donor T cells and induce deletion of GVH reactive T cells. This bi-directional clonal deletion establishes central tolerance (**Figure 4**). Yet central tolerance is not enough to allow immune suppression drug withdrawal without the risk of HVG (organ graft rejection) and GVH reactions. This is because mixed chimerism following low intensity host conditioning implies there are residual long living host post thymic T cells in the recipient that can mediate organ transplant rejection. Likewise, there are post thymic donor T cells that accompany the hematopoietic graft and that may persist perhaps even for decades and provide GVH reactions. Therefore, it is likely that peripheral mechanisms that help control bi- directional alloreactivity may also be needed. The Stanford protocols induced persistent mixed chimerism in the majority of recipients of HLA matched and mismatched recipients and consequently it is fair to assume based on current paradigms, that central tolerance was achieved (85–88). In the HLA matched setting, and after 6-months of persistent mixed chimerism, immune suppression drug withdrawal resulted in immune suppression independent continued mixed chimerism in about half of patients and a loss of chimerism in half (85–88). Whether mixed chimerism persisted or not after the complete withdrawal of immune suppression medication, kidney allograft rejection episodes were not observed with follow up extending to beyond 12 years (85–88).

These findings suggest that in the HLA matched setting, 6-months of persistent mixed chimerism may be a sufficient condition to induce long-lasting central tolerance that can control HVG alloreactivity and prevent graft rejection. The contribution and need for peripheral regulation in this setting is less defined. In a few instances however, patients who had had persistent mixed chimerism and subsequently lost the donor cells after immune suppression drug withdrawal, years later had an acute rejection episode. This suggests that central tolerance may not be a permanent state in a few patients or that peripheral regulation was lost.

In the HLA mismatched Stanford tolerance protocol low dose single drug immune suppression was required for persistent mixed chimerism as complete drug withdrawal resulted in loss of chimerism that was associated with acute rejection episodes (86, 87). This implied that in the HLA mismatched setting the development of ‘central tolerance’ may be imperfect. Alternatively, there may also be more need for peripheral immune regulation to prevent HVG alloreactivity mediated by long living post thymic host T cells not eradicated by TLI-ATG. In the newest iteration of the Stanford HLA mismatched protocol a single and very low dose of TBI (0.4-0.8 Gy) will be substituted for the last dose of TLI to provide additional host T cell depletion (decrease HVG reactions) and improve the levels of mixed chimerism; higher levels of chimerism within the first 6 months are expected to improve its stability even after immune suppression drug withdrawal. In a recently completed clinical trial in cancer patients TLI-ATG with a single very low dose of TBI host conditioning resulted in improved early chimerism without the toxicity associated with 2 Gy of TBI (https://clinicaltrials.gov/ct2/show/NCT03734601).

## Summary and Conclusions

The key observations of the link between chimerism and transplant tolerance were initially developed in rodents, and then studied in large laboratory animals and humans. Whereas stable mixed chimerism and organ graft acceptance without IS drugs was achieved in most MHC mismatched models of tolerance induction in rodents, it has been achieved only in MHC matched large animals and humans at present. Stable mixed chimeras showed specific immune unresponsiveness in both the HvG and GvH directions. Stable complete chimerism was achieved in MHC mismatched laboratory animals and humans given HCT as treatment for hematologic malignancies or for withdrawal of IS drugs with organ transplant acceptance. In the latter complete chimeric recipients, kidney graft acceptance was based on self-tolerance rather than HvG tolerance. Safety issues associated with a protocol that resulted in complete chimerism remain a clinical concern (**Table 1**) and included severe reductions in neutrophils and platelets due to the intensity of conditioning, and the increased risk of severe GVHD. Going forward, and as always, the successful wide-spread acceptance and adaptation of a cell-based immune suppression drug withdrawal protocol to other centers and to patients in-need considers the balance between the safety of the procedures, associated morbidities, the long-term risks, and the ability to significantly reduce and/or completely withdraw drug.

## Author Contributions

Both authors contributed to the article and approved the submitted version.

## Funding

This work was supported by the National Institute of Health, grant PO1HL075462 and R01 1085024, and the California Institute for Regenerative Medicine, grant Clin2-09439.

## Conflict of Interest

RL and SS are scientific founders of Medeor Therapeutics. SS is a consult to Medeor Therapeutics.

## Publisher’s Note

All claims expressed in this article are solely those of the authors and do not necessarily represent those of their affiliated organizations, or those of the publisher, the editors and the reviewers. Any product that may be evaluated in this article, or claim that may be made by its manufacturer, is not guaranteed or endorsed by the publisher.
